# Complex/cryptic *EWSR1::FLI1/ERG* Gene Fusions and 1q Jumping Translocation in Pediatric Ewing Sarcomas

**DOI:** 10.3390/genes14061139

**Published:** 2023-05-24

**Authors:** Ying S. Zou, Laura Morsberger, Melanie Hardy, Jen Ghabrial, Victoria Stinnett, Jaclyn B. Murry, Patty Long, Andrew Kim, Christine A. Pratilas, Nicolas J. Llosa, Brian H. Ladle, Kathryn M. Lemberg, Adam S. Levin, Carol D. Morris, Lisa Haley, Christopher D. Gocke, John M. Gross

**Affiliations:** 1 Johns Hopkins Genomics, Baltimore, MD 21205, USAjmurry3@jhu.edu (J.B.M.);; 2 Department of Pathology, Johns Hopkins University School of Medicine, Baltimore, MD 21205, USA; 3 Cytogenetics Laboratory, Johns Hopkins Medicine, Baltimore, MD 21205, USA; 4 Biotechnology, Johns Hopkins University, Baltimore, MD 21205, USA; akim140@jhu.edu; 5 Division of Pediatric Oncology, Sidney Kimmel Comprehensive Cancer Center, Johns Hopkins University, Baltimore, MD 21205, USA; cpratil1@jhmi.edu (C.A.P.); nllosa1@jhmi.edu (N.J.L.); bladle@jhmi.edu (B.H.L.); klember1@jhmi.edu (K.M.L.); 6 Department of Orthopaedic Surgery, Johns Hopkins University School of Medicine, Baltimore, MD 21205, USA; alevin25@jhmi.edu; 7 Orthopaedic Surgery Service, Memorial Sloan Kettering Cancer Center, New York, NY 10065, USA; morrisc@mskcc.org

**Keywords:** Ewing sarcomas, *EWSR1::FLI1* gene fusions, *EWSR1::ERG* gene fusion, 1q jumping translocation, three-way translocation, cryptic translocation

## Abstract

Ewing sarcomas (ES) are rare small round cell sarcomas often affecting children and characterized by gene fusions involving one member of the FET family of genes (usually *EWSR1)* and a member of the ETS family of transcription factors (usually *FLI1* or *ERG*). The detection of *EWSR1* rearrangements has important diagnostic value. Here, we conducted a retrospective review of 218 consecutive pediatric ES at diagnosis and found eight patients having data from chromosome analysis, FISH/microarray, and gene-fusion assay. Three of these eight ES had novel complex/cryptic *EWSR1* rearrangements/fusions by chromosome analysis. One case had a t(9;11;22)(q22;q24;q12) three-way translocation involving *EWSR1::FLI1* fusion and 1q jumping translocation. Two cases had cryptic *EWSR1* rearrangements/fusions, including one case with a cryptic t(4;11;22)(q35;q24;q12) three-way translocation involving *EWSR1::FLI1* fusion, and the other had a cryptic *EWSR1::ERG* rearrangement/fusion on an abnormal chromosome 22. All patients in this study had various aneuploidies with a gain of chromosome 8 (75%), the most common, followed by a gain of chromosomes 20 (50%) and 4 (37.5%), respectively. Recognition of complex and/or cryptic *EWSR1* gene rearrangements/fusions and other chromosome abnormalities (such as jumping translocation and aneuploidies) using a combination of various genetic methods is important for accurate diagnosis, prognosis, and treatment outcomes of pediatric ES.

## 1. Introduction

Ewing sarcomas (ES) are rare small round cell sarcomas, most often arising in the long bones of pediatric patients, but may occasionally also arise within soft tissues and affect adults [1]. They behave aggressively clinically, with a survival rate of 30% for those with metastatic disease. ES is genetically characterized by balanced reciprocal chromosomal translocations in which a member of the FET gene family is fused with an ETS transcription factor. The t(11;22) reciprocal translocation is the most common translocation [2,3], which fuses the N-terminal transactivation domain of the Ewing sarcoma breakpoint region 1 protein (EWSR1) with the C-terminal DNA binding domain of the Friend leukemia integration 1 transcription factor (FLI1) [4]. The *EWSR1::FLI1* gene fusion occurs in approximately 85% of ESs, followed by *EWSR1::ERG* gene fusion occurring in approximately 5% of ESs, and other *EWSR1* fusions/rearrangements at a much lower frequency, all of which are cancer-specific chimeric transcription factors that enormously restore the transcriptome leading to tumorigenesis of ES [4,5,6,7]. Therefore, the detection of *EWSR1* gene fusions/rearrangements in ES is of great diagnostic value. 

Routine genetic techniques used for detecting *EWSR1* translocations/rearrangements in ES include conventional chromosome analysis [2,8], fluorescence in situ hybridization (FISH) [9,10], reverse transcriptase-PCR (RT-PCR) [10], and advanced molecular techniques such as NanoString nCounter TagSet chemistry [11], or RNA-sequencing-based methods [12]. These molecular methods are often needed to help support or exclude a diagnosis of ES. Conventional chromosome analysis can provide structural and numerical chromosome abnormalities at a single cell level with a 5–10 Mb resolution. *EWSR1* break-apart FISH is a widely used technique for detecting *EWSR1* gene rearrangements without prior knowledge of the gene fusion partners. While dual-color dual-fusion *EWSR1::FLI1* FISH is specifically designed to detect the *EWSR1::FLI1* gene fusion, the NanoString nCounter assay/RNA-based method is a high-throughput hybridization technique that can be customized to test for many fusion transcripts in a single assay using RNA from formalin-fixed, paraffin-embedded material [11,12]. 

Complex and cryptic *EWSR1* gene rearrangements/fusions in ES have been reported mainly as individual case reports, and their frequencies in newly diagnosed ES patients at a routine cytogenetic laboratory are not well-recognized. Here, we performed a retrospective review of consecutive ES pediatric patients at initial diagnosis who had available data from conventional chromosome analysis, FISH/SNP microarray, and gene fusion assay. We found eight ES pediatric patients, and all patients in this study had complex karyotypes with various aneuploidies, structural chromosome abnormalities, and positive *EWSR1* FISH and gene fusion assay. Three of eight patients in this study cohort had novel complex and cryptic *EWSR1* rearrangements/fusions by conventional chromosome analysis. Two patients had cryptic *EWSR1* rearrangements/fusions, and one had a complex three-way translocation. With the emerging family of non-Ewing undifferentiated small round cell sarcomas of bone and soft tissue, including sarcomas with *CIC*-rearrangements, *BCOR*-alterations, and *EWSR*1-non *ETS* fusions, recognition of these complex and cryptic *EWSR1* fusions/rearrangements in ES is both diagnostically and prognostically valuable and often requires a multimodal molecular approach [13,14].

## 2. Materials and Methods

We performed a retrospective review of consecutive ES pediatric patients with new diagnoses who had available data from conventional chromosome analysis, FISH/SNP microarray, and gene fusion assay. From 1 January 1998 to 31 March 2023, 218 patients were referred to our hospital due to ES and had routine diagnostic procedures, such as morphologic evaluation, immunohistochemistry, conventional chromosome analysis, FISH/SNP microarray, and/or gene fusion assay. Only newly diagnosed pediatric ES patients concurrently having complete data from conventional chromosome analysis, FISH/SNP microarray, and gene fusion assay were selected for this study. Classification by standard ES practice and delineated by the World Health Organization was based on clinical, morphologic, immunophenotypic, and cytogenetic/molecular genetic features.

Conventional chromosome analysis/karyotyping (G-banded chromosome studies) was performed using standard techniques. A minimum of 20 metaphase cells were analyzed from tumor cultures. The abnormal karyotypes were described using the International System for Human Cytogenetic Nomenclature (ISCN 2020). Chromosome diagrams were drawn using the CyDAS package (http://www.cydas.org/, accessed on 1 April 2023).

Fluorescence in situ hybridization (FISH) was performed on interphase nuclei and metaphase cells using break-apart probes of *EWSR1* (Abbott Molecular, Inc., Des Plaines, Cook, IL, USA), dual-color, double-fusion probes of *EWSR1::FLI1* (Empire Genomics, Inc., Williamsville, New York, NY, USA), and sub-telomere probes for 4qter, 11qter, and 22qter (Oxford Gene Technology, Inc., Begbroke Science Park, Kidlington, UK) according to the manufacturer’s protocol as previously described [11,15]. A total of 200 interphase nuclei and 20 metaphase cells per probe were visually evaluated with fluorescence microscopy by two technologists scoring blinded from each other using a Zeiss Axioscope system (Carl Zeiss Microscopy, LLC, Oberkochen, 73447, Germany).

For a NanoString gene fusion panel, 300 ng RNAs from the formalin-fixed paraffin-embedded (FFPE) tumor specimens were used for the NanoString gene fusion assay in the present study following the manufacturer’s protocol as previously described [11]. 

Whole-genome SNP microarray was performed with DNA extracted from remnant fixed cell pellet using the Qiagen Puregene protocol or tumor specimens as previously described [11,15]. B-allele frequency and LogR signal intensities were used to examine and identify potentially pathogenic genomic imbalance regions. 

Immunohistochemistry was performed on the FFPE tumor specimens using the antibodies of CD99, desmin, myogenin, and NKX2.2 according to the manufacturer’s protocol. 

## 3. Results

### 3.1. Summary of the Patient Cohort in This Study

From a retrospective review of two-hundred eighteen consecutive ES pediatric patients at new diagnosis, we found fifteen ES pediatric patients who had available data from conventional chromosome analysis and FISH. Among these specimens, nine consecutive specimens from eight newly diagnosed pediatric ES patients simultaneously had complete data from conventional chromosome analysis, FISH/SNP microarray, and gene fusion assay (see Table 1). 

All patients in this study had abnormal karyotypes with various aneuploidies, structural chromosome abnormalities, and positive *EWSR1* FISH and gene fusion assay. Seven patients had *EWSR1::FLI1* gene fusions, and one had *EWSR1::ERG* gene fusion. Over one-third (patient IDs 3, 4, and 8 in Table 1) in our cohort had novel complex and cryptic *EWSR1* rearrangements/fusions by conventional chromosome analysis. One case had a complex three-way translocation and 1q jumping translocation (patient ID 3 in Table 1). Two cases had cryptic *EWSR1* rearrangements, including one with a cryptic three-way translocation leading to an *EWSR1::FLI1* fusion (patient ID 4 in Table 1), and the other had a cryptic *EWSR1::ERG* fusion on an abnormal chromosome 22 (patient ID 8 in Table 1). All eight patients in this study had various aneuploidies, with a gain of chromosome 8 (75%) being the most common and followed by gains of chromosome 20 (50%), chromosome 4 (37.5%), chromosome 2 (25%), chromosome 5 (25%), and chromosome 16 (25%), respectively.

### 3.2. Patient 3

**Figure 1:**
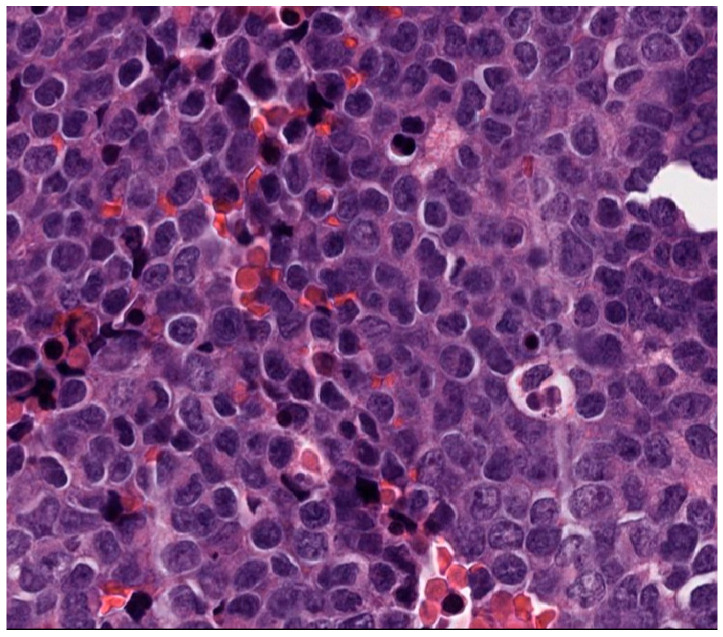
Histology of a humerus lesion in patient 3. The histologic evaluation shows monotonous small round blue cells with fine chromatin and scant amphophilic cytoplasm.

A male pediatric patient presented with right arm pain, swelling, and neurovascular compromise leading to numbness, weakness, paresthesia, shoulder pain with movement, and decreased appetite, concerning for oncological process. Magnetic resonance imaging (MRI) of the right arm extremity demonstrated a right humeral mass, and computed tomography (CT) of the chest demonstrated innumerable bilateral solid pulmonary nodules consistent with pulmonary metastases. He underwent a biopsy of the right humerus lesion, which revealed a monotonous round blue cell sarcoma with fine chromatin (Figure 1) that strongly expressed NKX2.2 and CD99, whereas desmin and myogenin were negative, supporting the diagnosis of ES.

**Figure 2:**
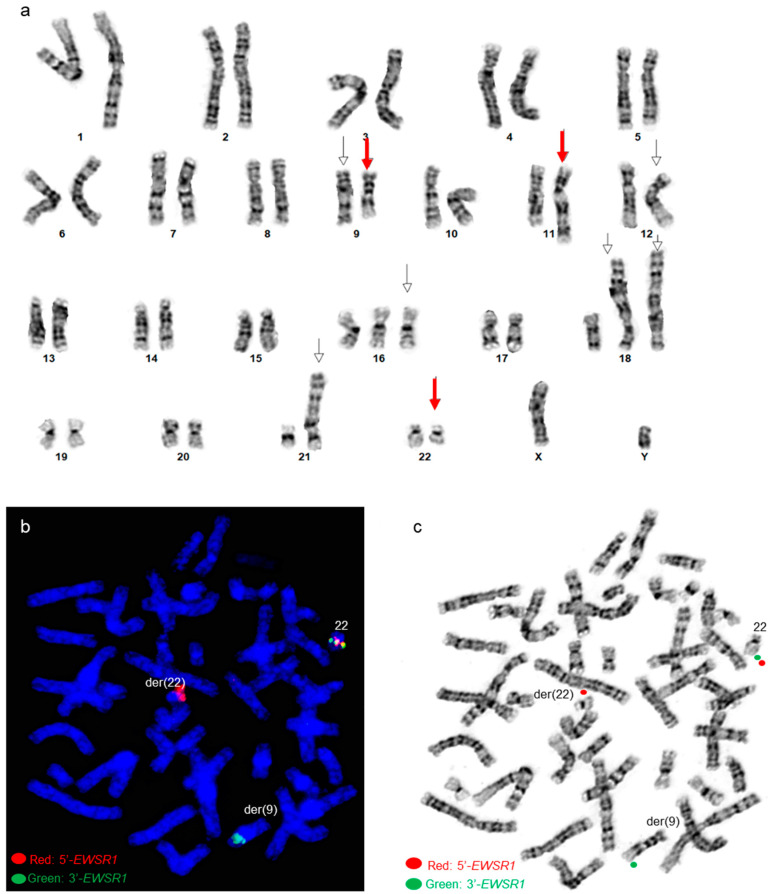
Cytogenetic analysis of patient 3 (**a**) A karyogram of 48,XY, del(9)(q34), t(9;11;22) (q22;q24;q12),add(12)(q24.1),+16,+18,der(18)t(1;18)(q12;p11.3)x2,der(21)t(1;21)(q12;p11.2). Red arrows point to the t(9;11;22) three-way translocation, and other arrows point to other abnormalities (**b**) Post-G-banded metaphase FISH using *EWSR1* break-apart probe shows *EWSR1* rearrangements. Green signals correspond to the 3′ portion of the *EWSR1* gene on the derivative chromosome 9, and red signals correspond to the 5′ portion of *EWSR1* remaining on the derivative 22. (**c**) *EWSR1* FISH signals in the G-banded metaphase cell.

By chromosome analysis, the right humerus ES tumor had an abnormal karyotype 48,XY,del(9)(q34),t(9;11;22)(q22;q24;q12),add(12)(q24.1),+16,+18,der(18)t(1;18)(q12;p11.3)x2,der(21)t(1;21)(q12;p11.2)[3]/49,sl,+13[6]/50,sdl,+20[11], which included a complex three-way translocation t(9;11;22)(q22;q24;q12), a 1q jumping translocation, various trisomies, and structural chromosomal abnormalities (Figure 2a). Using an *EWSR1* break-apart probe set, FISH revealed *EWSR1* rearrangements with 3′ *EWSR*1 on the derivative chromosome 9 involved in the three-way translocation (Figure 2b,c). 

**Figure 3:**
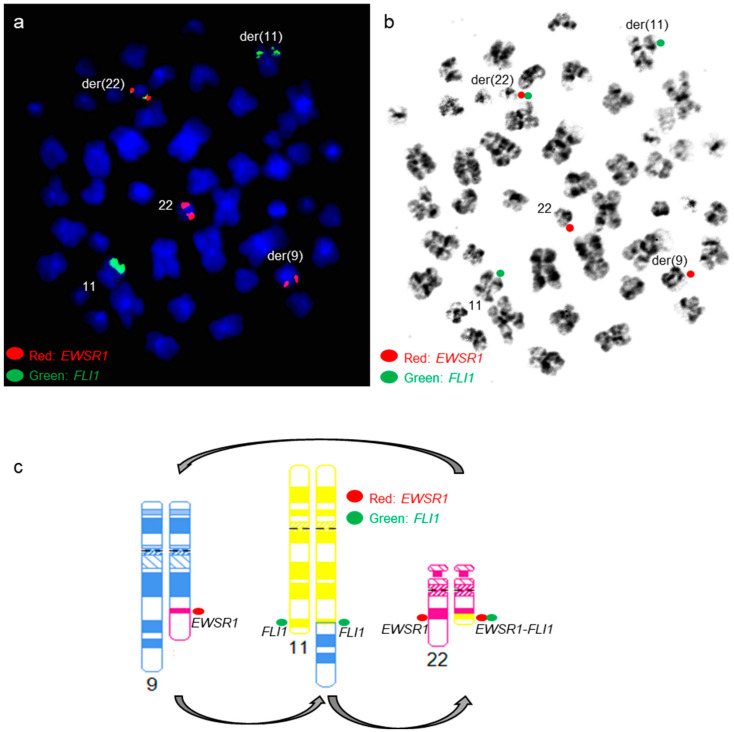
*EWSR1::FLI1* FISH analysis in patient 3 (**a**,**b**) Metaphase FISH using dual-color dual-fusion *EWSR1-FLI1* probe set shows *EWSR1::FLI1* fusion on the derivative chromosome 22 involved in the t(9;11;22)(q22;q24;q12) three-way translocation. Green signals are for the *FLI1* gene, and red signals are for the *EWSR1* gene. (**b**) FISH signals in the G-banded metaphase cell. (**c**) Diagrammatic representation of the complex three-way chromosomal rearrangement.

An *EWSR1::FLI1* fusion was present on the derivative chromosome 22 (der(22)) using a dual-color dual-fusion *EWSR1::FLI1* probe-set (Figure 3).

**Figure 4:**
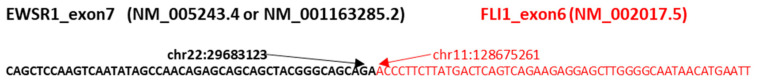
Visualization of the *EWSR1::FLI1* fusion identified by the NanoString gene fusion assay in patient 1. The genomic translocation breakpoints were mapped to chr22:29,683, 123 (exon 7) for *EWSR1* and chr11:128,675,261 (exon 6) for *FLI1*.

NanoString gene fusion panel revealed an EWSR1*::FLI1* fusion involving exon 7 of *EWSR1* and exon 6 of *FLI1* (Figure 4).

**Figure 5:**
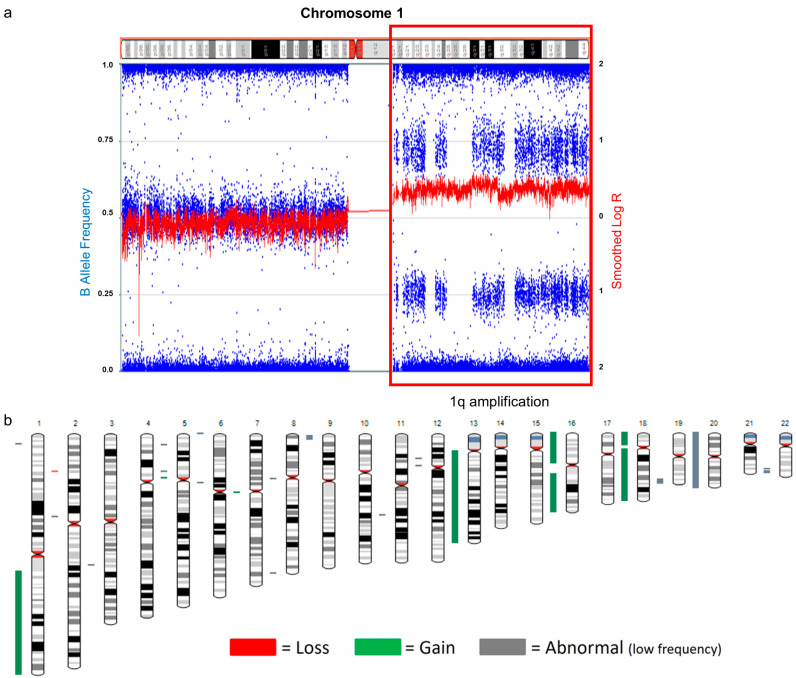
SNP microarray of patient 3. (**a**) Gain of 1q (arr[GRCh37] 1q12q44(142,632,577-249,218,992x1, shown in red box) by copy number plot based on LogR signal intensities (red line) and B-allele frequency plots (blue dots). (**b**) Whole-genome view of SNP microarray data.

SNP microarray revealed a high-level gain of chromosome 1q (Figure 5a) and a gain of chromosomes 13, 16, 18, and 20 (Figure 5b).

He was treated with standard multi-agent chemotherapy, which was tolerated without significant complications, and radiation to the humerus and lungs as definitive local control. Recurrent pulmonary metastatic disease was detected at 4 months after completion of treatment, and he was deceased 15 months after diagnosis due to respiratory failure as a result of widespread progressive pulmonary metastatic ES. 

### 3.3. Patient 4

A male pediatric patient presented with right knee pain and swelling. An MRI showed a right femoral diaphysis lesion, with subsequent biopsy showing a monotonous small round blue cell sarcoma with fine chromatin and scant amphophilic cytoplasm. The tumor was strongly and diffusely positive for NKX2.2 (nuclear) and CD99 (membranous) by immunohistochemistry, and a diagnosis of ES was rendered. A concurrent bone marrow biopsy did not reveal evidence of metastatic disease. A positron emission tomography (PET)/CT did not reveal evidence of distant disease.

**Figure 6:**
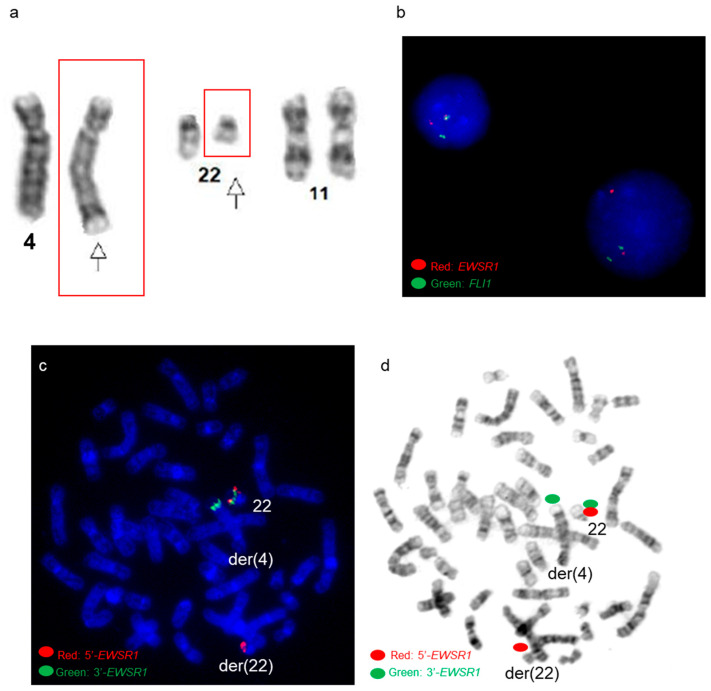
Cytogenetic analysis of patient 4 (**a**) Partial karyogram shows chromosomes 4, 11, and 22. Arrows point to the t(4;22)(q35;q12) reciprocal translocation by chromosome analysis. (**b**) FISH using *EWSR1* break-apart probe shows *EWSR1* rearrangements in interphase cells. (**c**,**d**) FISH using *EWSR1* break-apart probe shows *EWSR1* rearrangements in the G-banded metaphase cell. Green signals correspond to the 3′ portion of the *EWSR1* gene on the derivative chromosome 4, and red signals correspond to the 5′ portion of *EWSR1* remaining on the derivative chromosome 22. (**d**) *EWSR1* FISH signals in the G-banded metaphase cell.

By chromosome analysis, the right femoral ES had an abnormal karyotype of 46,XY,t(4;22)(q35;q12),+8,-10,+12,-16[3]/50,sl,+4,+8,+10,+16[6]/46,XY [11], which included a t(4;22)(q35;q12) reciprocal translocation (Figure 6a), tetrasomy 8, and other aneuploidies. FISH using an *EWSR1* break-apart probe set identified *EWSR1* rearrangement in 96% of interphase nuclei (Figure 6b), with the 3′ *EWSR1* locus on the derivative chromosome 4 involved in the 4;22 translocation (Figure 6c,d).

**Figure 7:**
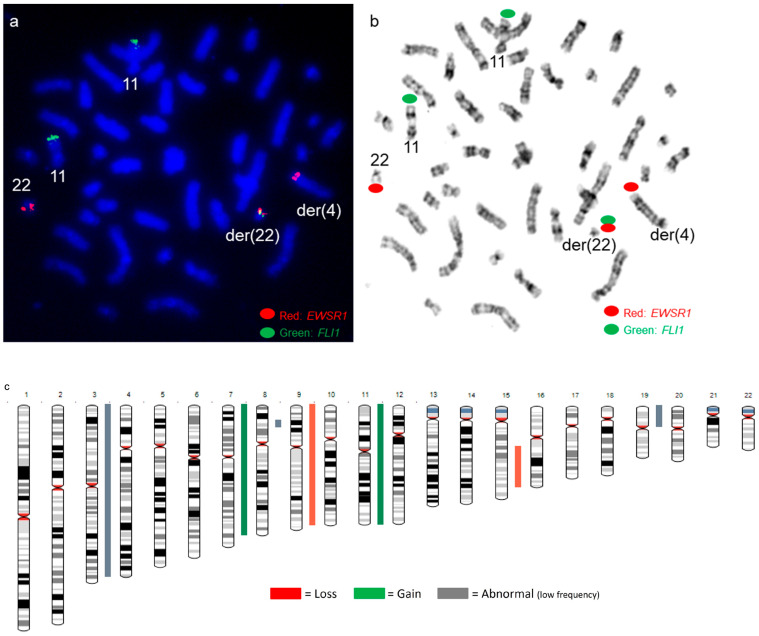
*EWSR1::FLI1* FISH and SNP microarray analysis in patient 4 (**a**,**b**) Metaphase FISH using dual-color dual-fusion *EWSR1-FLI1* probe set shows *EWSR1::FLI1* fusion on the derivative chromosome 22 involved in a cryptic t(4;11;22) three-way translocation. Green signals are for the *FLI1* gene, and red signals are for the *EWSR1* gene. (**b**) FISH signals in the G-banded metaphase cell. (**c**) Whole-genome view of SNP microarray data.

An *EWSR1::FLI1* fusion was present on the derivative chromosome 22 by a dual-color dual-fusion *EWSR1::FLI1* probe-set (Figure 7a,b). *FLI1* FISH signals were present on both chromosomes 11 (Figure 7a,b), and SNP microarray revealed normal copy numbers for chromosomes 11 and 22, including the *EWSR1* and *FLI1* genes (Figure 7c), suggesting the presence of a cryptic t(4;11;22)(q35;q24;q12) three-way translocation.

**Figure 8:**
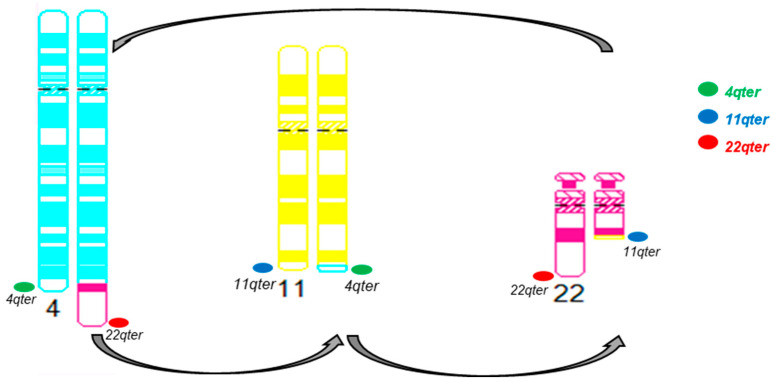
Diagrammatic representation of the cryptic three-way translocation by sub-telomere FISH for chromosomes 4qter, 11qter, and 22qter.

Sub-telomere FISH for chromosomes 4qter, 11qter, and 22qter was performed, and the FISH signal patterns further supported the presence of a cryptic t(4;11;22)(q35;q24;q12) three-way translocation (Figure 8).

**Figure 9:**
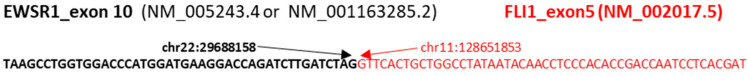
Visualization of the *EWSR1::FLI1* fusion identified by the NanoString gene fusion assay. The genomic translocation breakpoints were mapped to chr22:29,688,158 (exon 10) for *EWSR1* and chr11:128,651,853 (exon 5) for *FLI1*.

NanoString gene fusion panel revealed an *EWSR1::FLI1* fusion involving exon 10 of *EWSR1* and exon 5 of *FLI1* (Figure 9).

SNP microarray revealed copy number variants involving chromosomes 4, 8, 10, 12, and 16 (Figure 7c), consistent with the chromosome findings.

He was treated with standard multi-agent chemotherapy, and a significant decrease in the tumor size was seen after six neoadjuvant cycles of chemotherapy, which was well-tolerated. The patient underwent wide excision of the femur ES which, on pathologic review, demonstrated a significant treatment response with tumor necrosis of 99% and negative surgical margins. He completed adjuvant chemotherapy and is doing well, without evidence of recurrent disease.

### 3.4. Patient 8

A male pediatric patient initially presented with left-sided back and flank pain that started as dull and acutely worsened. He also developed a cough and lost several pounds. A chest CT showed a mass arising from the left posterior sixth rib with a questionable periosteal reaction of the seventh rib. He subsequently underwent a core needle biopsy of the lesion that showed a monotonous small round blue cell sarcoma with fine chromatin and scant amphophilic cytoplasm. The tumor was strongly positive for NKX2.2 (nuclear) and CD99 (membranous) by immunohistochemistry, and a diagnosis of ES was rendered. A concurrent bone marrow biopsy and full body PET/CT did not reveal evidence of metastatic disease.

**Figure 10:**
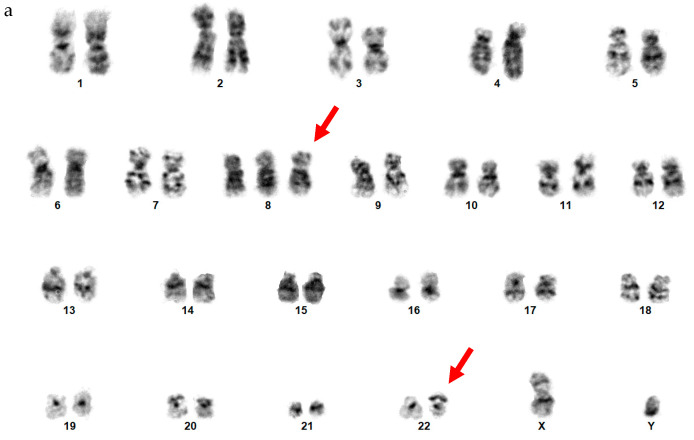

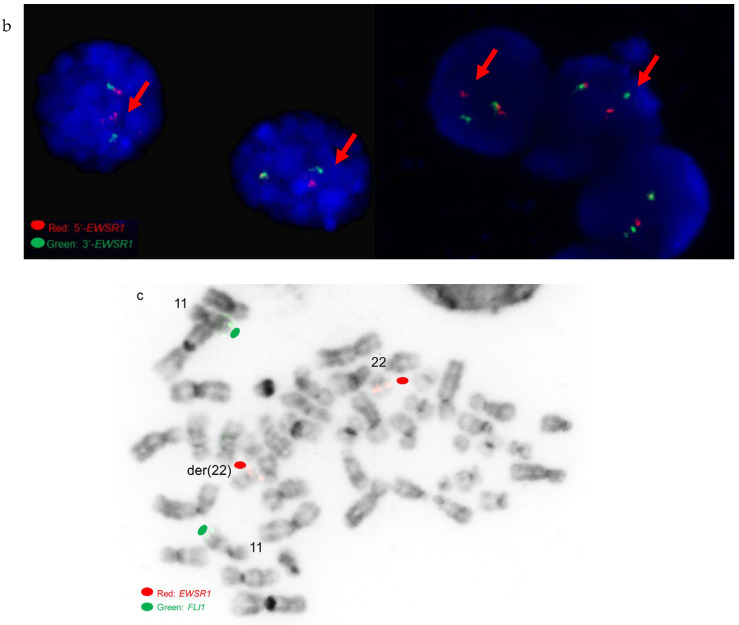
Cytogenetic analysis of patient 8 (**a**) A karyogram of 47,XY,+8,der(22)add(22) (p11.2)del(22)(q12). Red arrows point to trisomy 8 and the derivative chromosome 22. (**b**) Interphase FISH using *EWSR1* break-apart probe shows *EWSR1* rearrangements. Red arrows point to the cells with *EWSR1* gene rearrangements. (**c**) FISH using an EWSR1/FLI1 probe-set shows EWSR1 and FLI1 FISH signals in an inverted 4′,6-diamidino-2-phenylindole (DAPI)-banded metaphase cell.

By chromosome analysis, the left sixth rib ES had an abnormal karyotype of 47,XY,+8,der(22)add(22)(p11.2)del(22)(q12)[3]/46,XY[7], which included trisomy 8 and an abnormal chromosome 22 with additional material of unknown origin on the short arm of chromosome 22 and a terminal deletion of the long arm of chromosome 22 involving 22q12 band based on a suboptimal conventional chromosome analysis (Figure 10a).

By FISH, the left sixth rib ES had *EWSR1* rearrangements in 64% of interphase nuclei analyzed using an *EWSR1* break-apart probe set (Figure 10b). A subsequent FISH using a dual-color dual-fusion *EWSR1::FLI1* probe-set was negative for an *EWSR1::FLI1* fusion, both *EWSR1* genes were present on chromosomes 22 (a normal chromosome 22 and an abnormal derivative chromosome 22), and both *FLI1* FISH signals were present on both chromosomes 11 by metaphase FISH analysis (Figure 10c). NanoString gene fusion panel revealed an *EWSR1::ERG* fusion involving exon 7 of *EWSR1* and exon 9 of *ERG*.

He was treated with standard neoadjuvant multi-agent chemotherapy and then underwent resection of the tumor with a portion of the sixth rib where ES arose the seventh rib, and a small portion of the fifth rib. Tumor necrosis was approximately 50% on examination of the resection specimen. He completed adjuvant chemotherapy and is doing well without evidence of recurrence.

## 4. Discussion

As a pediatric bone tumor, ES often has *EWSR1* rearrangements/fusions, mainly *EWSR1::FLI1* fusions, frequently caused by reciprocal translocations between chromosomes 11 and 22. However, rare cases of complex three-way or four-way translocations and cryptic rearrangements/fusions leading to unusual *EWSR1::FLI1* and *EWSR1::ERG* gene fusions in ES have been reported [2,8,16,17,18,19]. Here, we performed a retrospective review of newly diagnosed ES patients. In this study, we found three out of eight patients (37.5%) harbor novel complex and cryptic *EWSR1* rearrangements/fusions in pediatric ES patients.

For complex translocations involving chromosomes 9, 11, and 22, two ES cases have been described previously [16,17]. One had a three-way translocation with t(9;?11;22) (p?24;?q24;q12) [16], and the other had a four-way translocation with t(9;11;22;13) (p22;q24;q12;q22) [17]. Both ES cases had breakpoints involving the short arm of chromosome 9 at either the 9p22 band or a possible 9p24 band. Our 9;11;22 three-way translocation had a breakpoint involving the long arm of chromosome 9 at the 9q22 band, which is different from previously reported 9;11;22 translocation cases.

Relevant to the second case highlighted, a t(4;22)(q35;q12) reciprocal translocation, it has previously been reported, by conventional chromosome analysis, in an extra-skeletal soft tissue (the distal aspect of right thigh) ES in a patient who presented with pulmonary metastases and paraplegia due to involvement of the spine [20]. However, FISH or other molecular diagnostic methods for an *EWSR1::FLI1* fusion was not performed [20]. Interestingly, a t(4;22)(q35;q12) translocation has also been reported in a case of embryonal rhabdomyosarcoma and results in the fusion of the *EWSR1* gene with the *DUX4* gene [21]. In our case, the t(4;22)(q35;q12) reciprocal translocation involved an *EWSR1* rearrangement and *EWSR1::FLI1* fusion on the derivative chromosome 22. The *EWSR1::FLI1* metaphase FISH signal pattern, normal copy number for *EWSR1* and *FLI1* genes by SNP microarray, and sub-telomere FISH signal patterns support the presence of a possible cryptic t(4;11;22) three-way translocation instead of a potential *FLI1* insertion into the derivative chromosome 22. For a t(4;11;22) three-way translocation, at least two ES cases have been described previously [18,19]. Both cases had breakpoints involving the long proximal arm of chromosome 4 at either the 4q21 band or the 4q25 band [18,19]. The t(4;22)(q35;q12) reciprocal translocation in our case involved the distal (near sub-telomere) region of the long arm of chromosome 4 at the 4q35 band, resulting in a cryptic t(4;11;22)(q35;q24;q12) three-way translocation by conventional chromosome analysis. The previously reported ES case with a t(4;22)(q35;q12) reciprocal translocation by conventional chromosome analysis [20] may also have a cryptic t(4;11;22)(q35;q24;q12) three-way translocation leading to an *EWSR1::FLI1* fusion. Therefore, performing FISH or gene fusion-based assays for an *EWSR1::FLI1* fusion in these ES cases is vital to rule out a cryptic three-way translocation or other complex rearrangements.

Cryptic and complex rearrangements resulting in *EWSR1::ERG* fusions have been reported previously [22,23,24]. Most of these have abnormalities on chromosome 22, such as a derivative chromosome 22 with a breakpoint on 22q12 and a suggestion of *EWSR1* gene rearrangements, but do not have obvious t(21;22)(q22;q12) reciprocal translocation leading to *EWSR1::ERG* fusions. These cryptic *EWSR1::ERG* fusions are commonly present on derivative chromosome 22 via an inversion-insertion mechanism (by either the 5′ fraction of the *EWSR1* gene or the 3′ fraction of the *EGR* gene inverted and inserted to develop the fusion) [22]. Cryptic *EWSR1::ERG* fusions inserted into chromosome 21 have been reported in at least two cases [23,24]. One case has a cryptic *EWSR1::ERG* fusion inserted into a complex three-way translocation t(1;21;7)(q25;q22.3;q22) involving the long arms of chromosomes 1, 7, and 21 [23]. Both cases have a cryptic *EWSR1::ERG* fusion inserted into the long arm of a derivative chromosome 21. Patient 8 in this study likely has an *EWSR1::ERG* fusion inserted into the long arm of a derivative chromosome 22. Since gene fusions can insert into different arms of various chromosomes when the classic t(11;22) or t(21;22) reciprocal translocation is not apparent in a karyotype, alternative methods such as FISH or a gene fusion assay could be performed to look for a variant or cryptic gene fusion in ES.

Most of the patients in our study contained complex karyotypes with various aneuploidies and structural chromosomal abnormalities. Although the gain of chromosome 8 is common and seems to be recurrent in ES, structural chromosomal abnormalities seem to be random, except gain of 1q. Two patients (patient IDs 3 and 6, 25% in this study) had a gain of 1q. Patient 3 had jumping translocations, and patient 6 had whole-arm translocations. Jumping translocations have been defined as nonreciprocal translocations involving a donor chromosome arm or chromosome segment fused to several different recipient chromosomes [25]. Jumping translocations involving 1q12–21 as the donor chromosome segment are referred to as 1q jumping translocations. 1q jumping translocations have been infrequently reported in patients with multiple myeloma, malignant lymphoproliferative disorders, and myeloid malignancies [26,27,28]. They result in 1q gain and possible loss of segments of the recipient chromosomes. In terms of recipient chromosomes, approximately 43% of reported 1q jumping translocations in myeloid malignancies occurred in the short arms of the five acrocentric chromosomes, and over one-third occurred in telomeric regions of chromosome arms [27]. The 1q jumping translocation, in our case, involved the short arm of an acrocentric chromosome 21 and the telomeric region of chromosome 18p. 1q gain has been identified as a secondary genetic alteration with a significant prevalence in ES, which was also markedly associated with relapse and poor overall and disease-free survival [29,30]. Although the large heterochromatic regions at 1q12 might be responsible for the frequent translocation breakpoint leading to the gain of the long arm of chromosome 1 [31,32], to the best of our knowledge, 1q jumping translocations have not been previously described in pediatric ES along with a complex three-way translocation.

## 5. Conclusions

In summary, we identified three novel complex and cryptic rearrangements leading to *EWSR1::FLI1* and *EWSR1::ERG* gene fusions with various breakpoints in our cohort of newly diagnosed pediatric ES patients. Two patients had either a cryptic three-way translocation or a cryptic insertion/complex rearrangement, which were not recognizable by conventional chromosome analysis. A high proportion of novel complex and cryptic rearrangements/fusions in ES patients add challenges to identifying these variants *EWSR1::FLI1* and *EWSR1::ERG* fusions within a routine clinical cytogenetic laboratory. We advocate an essential cytogenetic/molecular genetic approach, including the integration of conventional chromosome analysis, FISH, and gene fusion assays to increase the detection of variant rearrangements/fusions in ES patients. Furthermore, most ES patients in this study had a complex karyotype with both recurrent numerical and structural chromosomal abnormalities. Recognizing complex (three-way translocations) and cryptic (translocations/insertions/inversions) *EWSR1::FLI1* and *EWSR1::ERG* gene fusions along with other chromosome abnormalities (such as 1q jumping translocations, tetrasomy, aneuploidies, and structural chromosomal abnormalities) using a combination of various diagnostic methods is important for accurate diagnosis, prognosis, and treatment outcomes of pediatric Ewing sarcomas.

## Figures and Tables

**Table 1:** Summary of the study cohort.

ID	Ewing Sarcoma (Location)	Conventional Chromosome Analysis (Karyotype of Abnormal Clones)	FISH (Positive for)	Gene Fusion
1	L. chest wall	49,XX,+4,+8,t(11;22)(q24;q12),+20/96-99,slx2	*EWSR1*	*EWSR1::FLI*
2	R. iliac crest	49,XX,+5,+8,+8,t(11;22)(q24;q12)	*EWSR1*	*EWSR1::FLI*
3	R. humerus & metastatic lung	48,XY,del(9)(q34),t(9;11;22)(q22;q24;q12),add(12)(q24.1),+16, +18,der(18)t(1;18)(q11;p11.3)x2,der(21)t(1;21)(q11;p11.2)/ 49,sl,+13/50,sdl,+20	*EWSR1* & *EWSR1::FLI1*	*EWSR1::FLI*
4	R. femur	46,XY,t(4;22)(q35;q12),+8,-10,+12,-16/50,sl,+4,+8,+10,+16	*EWSR1* & *EWSR1::FLI1*	*EWSR1::FLI*
5	L. pubic bone	96-104,XXXX,add(1)(p12),i(1)(q10),+2,+2,+4,+4,+8,+8,+8,+8,+9, t(11;22)(q24;q12)x2,der(16)t(12;16)(q13;q24)x2,+20,+20	*EWSR1*	*EWSR1::FLI*
6	R. lung & metastatic lymph node	47,XX,+1,+1,der(1;15)(q10;q10)x2,add(2)(q35),del(5)(q31),t(11;22)(q24;q12), +15	*EWSR1*	*EWSR1::FLI*
7	L. femur	51,XX,+2,+5,+7,+8,t(11;22)(q24;q12),+20 at diagnosis	*EWSR1*	*EWSR1::FLI*
		51,XX,+2,+5,+7,+8,del(10)(q24q25),t(11;22)(q24;q12),+20 at follow-up	*EWSR1*	*EWSR1::FLI*
8	L. chest wall	47,XY,+8,der(22)add(22)(p11.2)del(22)(q12)	*EWSR1* *	*EWSR1::ERG*

L.: left; R.: right; *: This case is negative for *EWSR1::FLI1* by FISH.

## Data Availability

The dataset for the current study is available from the corresponding author upon reasonable request.
